# A Novel Non-invasive Model Based on GPR for the Prediction of Liver Fibrosis in Patients With Chronic Hepatitis B

**DOI:** 10.3389/fmed.2021.727706

**Published:** 2021-09-23

**Authors:** Rongrong Ding, Wei Lu, Xinlan Zhou, Dan Huang, Yanbing Wang, Xiufen Li, Li Yan, Weijia Lin, Shu Song, Zhanqing Zhang, Liang Chen

**Affiliations:** ^1^Department of Hepatobiliary Medicine, Shanghai Public Health Clinical Center, Fudan University, Shanghai, China; ^2^Department of Pathology, Shanghai Public Health Clinical Center, Fudan University, Shanghai, China; ^3^Department of Liver Disease, Shanghai Public Health Clinical Center, Fudan University, Shanghai, China

**Keywords:** CHB, liver fibrosis, type IV collagen, INR, GPR

## Abstract

**Background:** Some controversy remains regarding conventional serum indices for the evaluation of liver fibrosis. Therefore, we aimed to combine the existing index with other serum parameters to discriminate liver fibrosis stages in patients with chronic hepatitis B (CHB).

**Methods:** A total of 1,622 treatment-naïve CHB patients were divided into training (*n* = 1,211) and validation (*n* = 451) cohorts. Liver histology was assessed according to the Scheuer scoring scheme. All common demographic and clinical parameters were analyzed.

**Results:** By utilizing the results of the logistic regression analysis, we developed a novel index, the product of GPR, international normalized ratio (INR), and type IV collagen (GIVPR), to discriminate liver fibrosis. In the training group, the areas under the ROCs (AUROCs) of GIVPR, APRI, FIB-4, and GPR for significant fibrosis were 0.81, 0.75, 0.72, and 0.77, respectively; the AUROCs of GIVPR, APRI, FIB-4, and GPR for advanced fibrosis were 0.82, 0.74, 0.74, and 0.78, respectively; and the AUROCs of GIVPR, APRI, FIB-4, and GPR for cirrhosis were 0.87, 0.78, 0.78, and 0.83, respectively. Similar results were also obtained in the validation group. Furthermore, the decision curve analysis suggested that GIVPR represented superior clinical benefits in both independent cohorts.

**Conclusion:** The GIVPR constructed on GPR represents a superior predictive model for discriminating liver fibrosis in CHB patients.

## Background

Hepatitis B virus (HBV) infection is a serious public health problem. It is estimated that more than 350 million people are chronically infected worldwide (1). From 1990 to 2013, the mortality rate of liver cirrhosis and hepatocellular carcinoma caused by HBV infection increased by 33% worldwide (2). Based on the outcomes of patients who receive early diagnosis and effective antiviral therapy, the prognosis of CHB can be significantly improved even if the case is histologically advanced fibrosis or cirrhosis (3). Therefore, it is of great importance to assess the risk of early liver fibrosis in CHB patients.

Currently, the gold standard for the assessment of liver fibrosis is still liver biopsy. However, its limitations, such as its invasiveness, sampling errors, cost, intra- and inter-observer discrepancies, and the risk of potentially life-threatening complications, restrict its clinical application (4). Clinical practice requires simple operations or non-invasive and easy methods to diagnose liver inflammation, injury or fibrosis (5). The World Health Organization (WHO) guidelines recommend serologic biomarkers and FibroScan as useful non-invasive methods for evaluating CHB patients (6). However, several factors, including necroinflammatory activity, ascites, cost, and lack of skilled operators, may diminish the clinical use of FibroScan (6, 7). Serum biomarkers are particularly important in these methods because they do not require qualified staff and expensive equipment for evaluation (8). The WHO has recommended the aspartate aminotransferase (AST)-platelet ratio index (APRI) and fibrosis-4 (FIB-4) as non-invasive indices for CHB patients (6). The diagnostic value of these two indices in liver fibrosis has been widely studied, but their sensitivity and specificity are still controversial (9). Recently, a study by Lemonie et al. (10) suggested that the γ-glutamyl transpeptidase to platelet ratio (GPR) was more accurate than APRI or FIB-4, and this study was supported by several studies on Chinese subjects (11, 12). However, there were still a few inconsistent conclusions (13). Therefore, novel non-invasive serum calculations are still needed because the current biochemical markers do not have enough diagnostic accuracy to replace liver biopsy.

Serum collagen, especially type IV collagen, has been confirmed to be a useful, non-invasive marker for measuring the activity of this pathway at a single time point and has been shown to reflect prognosis and responses to a variety of chronic liver diseases (14). INR is a routine serological marker associated with liver function and essentially reflects the progression of liver diseases. Wu et al. reported that the INR was an independent factor for the prediction of significant fibrosis in patients with CHB (6, 15).

More efforts should be dedicated to pursuing simple, safe and reliable non-invasive diagnostic measures to stage liver fibrosis. In this study, we aimed to construct and validate a predictive index consisting of GPR, INR, and type IV collagen to reflect liver fibrosis simply and effectively in CHB patients.

## Methods

### Patients

Overall, between January 2014 and January 2021, we retrospectively screened 2,193 consecutive Chinese individuals with chronic hepatitis B who underwent liver biopsy and clinical examination at Shanghai Public Health Clinical Center, Fudan University. CHB was diagnosed when serum hepatitis B surface antigen (HBsAg) was persistently positive for more than 6 months (16). All the patients were >18 years old. Non-alcoholic fatty liver disease (NAFLD) was diagnosed as at least 5% biopsy-proven hepatic steatosis without significant alcohol consumption (17). The exclusion criteria were as follows: antiviral treatment history, coinfection with hepatitis C virus (HCV), hepatitis D virus (HDV), hepatitis E virus (HEV), or human immunodeficiency virus (HIV), significant alcohol consumption (>20 g/d), autoimmune hepatitis, hepatocellular carcinoma, decompensated cirrhosis, inadequate liver biopsy samples (<1.5 cm), and the use of warfarin.

**Figure 1:**
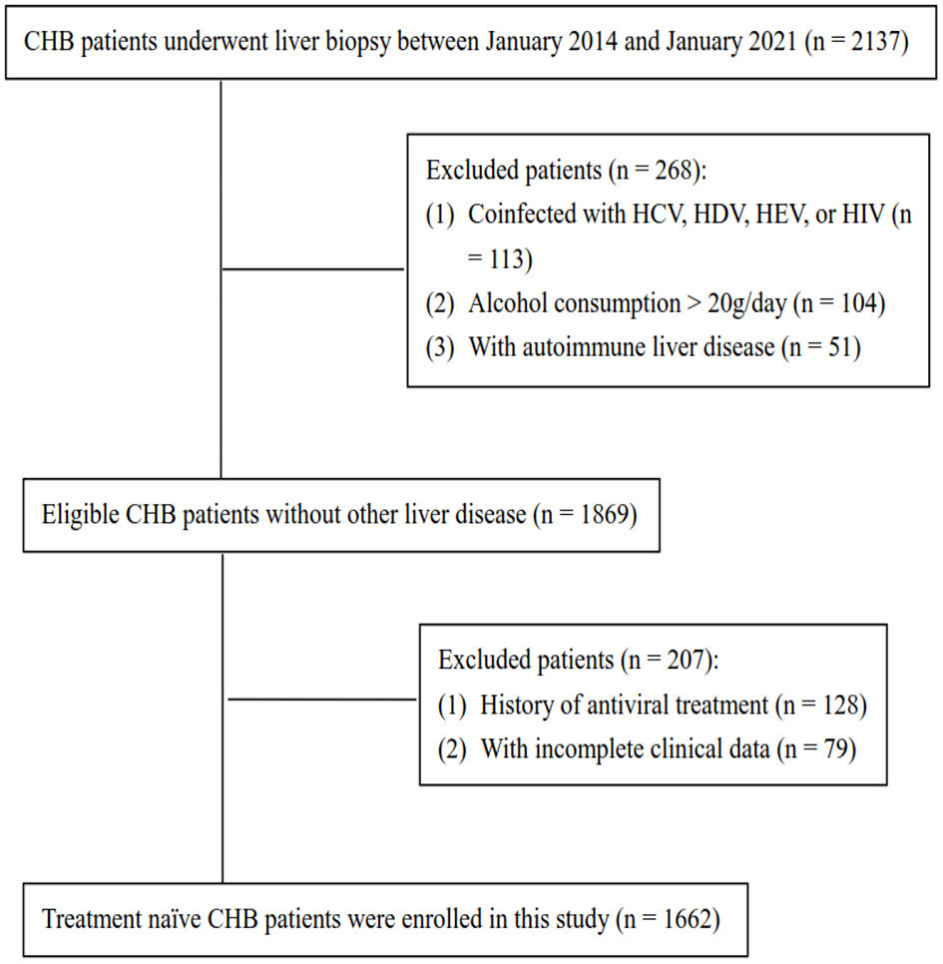
Flow diagram of this study population. CHB, chronic hepatitis B; HCV, hepatitis C virus; HDV, hepatitis D virus; HEV, hepatitis E virus; HIV, human immunodeficiency virus.

We summarized the flow diagram of the study population in Figure 1. After excluding patients with coinfection with HCV, HDV, HEV, or HIV (*n* = 113), alcohol consumption (>20 g/d) (*n* = 104), autoimmune hepatitis (*n* = 51), history of antiviral treatment (*n* = 128), and incomplete clinical data (*n* = 79), 1,662 treatment-naïve patients with CHB were included. The population was randomly divided into a training set (*n* = 1,211) and a validation set (*n* = 451) for model development and validation using SPSS software.

### Liver Biopsy

Percutaneous liver biopsy was performed using a 16 G needle under ultrasound guidance. Liver samples with a minimum length of 1.5 cm and at least 7 complete portal tracts were fixed in 10% formalin, embedded in paraffin, and stained with HE Masson's trichrome and reticulin for histological analysis. Liver histology was analyzed by two experienced pathologists who were blinded to other clinical and laboratory data and classified according to the Scheuer scoring system (18) as follows: S0 (no fibrosis), S1 (mild fibrosis without septa), S2 (moderate fibrosis with few septa), S3 (severe fibrosis with numerous septa without cirrhosis), and S4 (cirrhosis). In this study, liver fibrosis stage ≥S2 was defined as significant fibrosis, ≥S3 was defined as advanced fibrosis, and S4 was defined as cirrhosis. These definitions represent at minimum significant fibrosis and affect the management of patients in terms of treatment indications (16, 19).

### Laboratory Data

Fasting blood samples were obtained within a week of liver biopsy. Platelets and other blood cells were counted using a Sysmex-XT 4000i automated hematology analyzer. The international normalized ratio (INR) and other coagulation indices were measured using a STAR Max automatic coagulation analyzer. Alanine transaminase (ALT), aspartate aminotransferase (AST), alkaline phosphatase (ALP), γ-glutamyl transferase (GGT), hyaluronic acid, laminin, N-terminal propeptide of type III procollagen (PIIINP), type IV collagen, and other serum biochemical parameters were measured using an Architect C16000 automatic biochemical analysis system.

### Formulas

The formulas for APRI, FIB-4, and GPR are as follows: APRI = (AST (U/L)/ULN of AST)/platelet count (10^9^/L) × 100 (20); FIB-4 = (age (years) × AST (U/L))/(platelet count (10^9^/L) × (ALT (U/L))^1/2^) (21); GPR = (GGT (U/L)/ULN of GGT)/platelet count (10^9^/L) × 100 (10).

### Statistical Analysis

Statistical analysis was performed using IBM SPSS Statistics version 26.0 (SPSS Inc., Chicago, USA) and R 4.0.3 (http://www.R-project.org). Continuous variables are expressed as the mean ± standard deviation or median (interquartile range, IQR) and were compared using Student's *t*-test (for normally distributed continuous variables) or the independent Mann–Whitney *U*-test (for non-normally distributed continuous variables). Categorical variables are expressed as proportions and were compared by the chi-square test. Logistic regression models were used to assess the correlations between variables and liver fibrosis. The performances of the non-invasive markers for predicting liver fibrosis were assessed by receiver operating characteristic (ROC) curve analyses. The Delong *Z*-test was used to compare the AUROCs of the serum models. Decision curve analysis (DCA) was used to further evaluate the predictive performances. A two-sided *P* < 0.05 was considered statistically significant.

## Results

### Clinical Characteristics of the Study Population

A total of 1,662 treatment-naïve CHB patients who had undergone a liver biopsy were enrolled in the study, with median ages of 37 (31–45) and 37 (31–45) years in the training and validation sets, respectively. The clinical data of the studied groups are summarized in Table 1. Except for ALT, there were no statistically significant differences in other parameters between the training and validation sets. Additionally, 293 (24.2%) patients were in fibrosis stage S2, 138 (11.4%) were in S3, and 255 (21.1%) were in S4 in the training set, while 113 (25.1%) patients were in S2, 43 (9.5%) were in S3, and 77 (17.1%) were in S4 in the validation set.

**Table 1:** Clinical characteristics of studied patients with CHB.

**Variables**	**Training set (*****n*** **= 1,211)**	**Validation set (*****n*** **= 451)**	***P*** **-value**
Age, years	37 (31-45)	37 (31-45)	0.419
Male, *n* (%)	779 (64.3)	296 (65.6)	0.649
NAFLD, *n* (%)	120 (9.9)	57 (12.6)	0.109
**Serum parameters**			
logHBVDNA, IU/ml	5.15 (3.06-7.11)	5.28 (3.16-7.08)	0.584
ALT, U/L	48.00 (26.00-119.00)	54.00 (30.00-134.00)	0.041
AST, U/L	35.00 (23.00-71.00)	38.00 (24.00-79.00)	0.117
ALP, U/L	75.00 (62.00-93.00)	77.00 (63.00-97.00)	0.222
GGT, U/L	32.00 (18.00-67.00)	35.00 (19.00-73.00)	0.078
TBil, μmol/L	14.30 (10.20-19.90)	15.00 (10.50-21.00)	0.054
DBil μmol/L	5.40 (3.90-7.59)	5.60 (4.10-8.00)	0.100
Albumin, g/L	42.40 (39.62-45.09)	42.10 (39.30-45.00)	0.476
FBG, mmol/L,	4.90 (4.55-5.32)	4.95 (4.58-5.40)	0.305
TC, mmol/L	4.19 (3.68-4.85)	4.26 (3.67-4.90)	0.325
TG, mmol/L	0.96 (0.72-1.30)	0.99 (0.74-1.34)	0.184
HDL, mmol/L	1.34 (1.07-1.58)	1.27 (1.01-1.56)	0.059
LDL mmol/L	2.63 (2.14-3.17)	2.73 (2.17-3.18)	0.161
Urea, mmol/L	307.02 (253.48 0-362.49)	300.55 (251.90-366.70)	0.519
Creatinine,μmol/L	65.50 (53.99-74.81)	64.40 (54.30-74.78)	0.725
INR	1.05 (0.99-1.11)	1.04 (1.00-1.11)	0.958
APTT, s	38.40 (35.80-41.20)	38.50 (36.10-40.90)	0.695
Fibrinogen, g/L	2.45 (2.14-2.78)	2.45 (2.15-2.78)	0.963
WBC count, ×10^9^/L	5.27 (4.36-6.23)	5.21 (4.33-6.16)	0.528
Platelet count, ×10^9^/L	165.00 (131.00-203.00)	168.00 (132.00-201.00)	0.574
Neutrophils count, ×10^9^/L	2.87 (2.26-3.63)	2.82 (2.19-3.52)	0.263
Lymphocyte count, ×10^9^/L	1.75 (1.41-2.16)	1.80 (1.44-2.21)	0.146
Hyaluronic, ng/ml	60.20 (41.00-98.06)	59.82 (42.63-98.49)	0.543
Laminin, ng/ml	25.49 (18.53-38.98)	26.01 (17.79-39.78)	0.667
PIIINP, ng/ml	25.74 (17.95-38.07)	25.69 (18.15-39.27)	0.680
Type IV collagen, ng/ml	26.01 (20.11-36.51)	26.53 (19.47-38.69)	0.267
**Non-invasive indexes**			
APRI	0.59 (0.33-1.38)	0.64 (0.35-1.29)	0.058
FIB-4	1.28 (0.83-2.07)	1.28 (0.86-1.99)	0.187
GPR	0.40 (0.21-0.95)	0.44 (0.23-1.09)	0.115
**Liver pathology**			
Scheuer fibrosis stage (S0-1/S2/S3/S4)	525(43.4%)/293(24.2%)/ 138 (11.4%)/255(21.1%)	218 (48.3%)/113(25.1%)/43 (9.5%)/77(17.1%)	0.134
Scheuer activity grade (G0-1/G2/G3/G4)	667 (55.1%)/335(27.7%)/209(17.3%)/0	251(55.7%)/115(25.5%)/85(18.8%)/0	0.590

*NAFLD, non-alcoholic fatty liver disease; ALT, alanine transaminase; AST, aspartate aminotransferase; ALP, alkaline phosphatase; GGT, γ-glutamyl transpeptadase; TBil, total bilirubin; DBil, direct bilirubin; FBG, fasting blood glucose; TC, total cholesterol; TG, triglyceride; LDC, low-density lipoprotein; HDL, high-density lipoprotein; INR, international normalized ratio; APTT, activated partial thromboplastin time; PIIIP, N-Terminal procollagen III propeptide; APRI, AST to platelet ratio index; FIB-4, fibrosis-4; GPR, GGT to platelet ratio*.

### Development of the GIVPR Index in the Training Cohort

In the training cohort, a significantly increased odds ratio of stage S2–4 was associated with age, NAFLD, HBV DNA, ALT, AST, ALP, GGT, total bilirubin (TBil), direct bilirubin (DBil), albumin, total cholesterol (TC), triglyceride (TG), INR, activated partial thromboplastin time (APTT), white blood cells (WBC), neutrophils, platelets, hyaluronic, laminin, PIIINP, and type IV collagen. Multivariable analysis identified TBil, INR, platelets, and type IV collagen as independent predictors of significant liver fibrosis. Similarly, a significantly increased odds ratio of stage S4 was associated with sex, age, NAFLD, ALT, AST, ALP, GGT, TBil, DBil, albumin, TC, high-density lipoprotein (HDL), low-density lipoprotein (LDL), INR, APTT, WBC, neutrophils, lymphocytes, platelets, hyaluronic, laminin, PIIINP, and type IV collagen. Multivariable analysis identified ALP, INR, platelets, and type IV collagen as independent predictors of cirrhosis (Table 2). Thus, in addition to platelets, both INR and type IV collagen were independent predictors of significant fibrosis and cirrhosis (all *P* < 0.01).

**Table 2:** Variables associated with significant fibrosis and cirrhosis by logistic analysis in training cohort.

**Variables**	**Significant fibrosis (S2-4)**				**Cirrhosis (S4)**			
	**Univariate**		**Multivariate**		**Univariate**		**Multivariate**	
	**OR (95% CI)**	***P*** **-value**	**OR (95% CI)**	***P*** **-value**	**OR (95%CI)**	***P*** **-value**	**OR (95% CI)**	***P*** **-value**
Gender, male	0.71 (0.65-1.04)	0.251			0.57 (0.42-0.77)	<0.001		
Age	1.01 (1.00-1.02)	0.105			1.02 (1.01-1.03)	0.001		
NAFLD, yes vs. no	0.53 (0.36-0.78)	0.001			0.51 (0.27-0.89)	0.017		
LogHBVDNA, IU/ml	1.07 (1.02-1.13)	0.013			1.04 (0.97-1.11)	0.285		
ALT, U/L	1.00 (1.00-1.01)	<0.001			1.00 (1.00-1.00)	<0.001		
AST, U/L	1.01 (1.00-1.01)	<0.001			1.00 (1.00-1.00)	<0.001		
ALP, U/L	1.02(1.02-1.03)	<0.001			1.02 (1.01-1.02)	<0.001	1.01 (1.00-1.01)	0.007
GGT, U/L	1.02(1.01-1.02)	<0.001			1.01 (1.01-1.01)	<0.001		
TBil,μmol/L	1.02 (1.01-1.03)	<0.001	0.98 (0.97-0.99)	0.025	1.03 (1.02-1.04)	<0.001		
DBil, μmol/L	1.04 (1.03-1.06)	<0.001			1.03 (1.02-1.05)	<0.001		
Albumin, g/L	0.87 (0.85-0.90)	<0.001			0.81 (0.78-0.84)	<0.001		
FBS	0.96 (0.84-1.08)	0.483			1.04 (0.90-1.19)	0.620		
TC, mmol/L	0.78 (0.69-0.88)	<0.001			0.62 (0.53-0.74)	<0.001		
TG, mmol/L	0.76 (0.63-0.91)	0.004			0.96 (0.77-1.19)	0.682		
LDL, mmol/L	1.00 (0.98-1.03)	0.899			0.62 (0.51-0.74)	<0.001		
HDL, mmol/L	0.92 (0.71-1.21)	0.563			0.54 (0.38-0.76)	<0.001		
Creatinine, μmol/L	1.00 (0.99-1.01)	0.778			1.00 (0.99-1.01)	0.900		
Urea, mmol/L	1.00 (0.99-1.00)	0.216			1.00 (0.99-1.00)	0.978		
INR	2.22 (1.92-2.57)	<0.001	**1.69 (1.39-2.06)**	**<0.001**	2.60 (2.22–3.05)	<0.001	**1.70 (1.40-2.06)**	**<0.001**
APTT,	1.09 (1.06-1.13)	<0.001			1.04 (1.01-1.08)	0.009		
Fibrinogen, g/L	0.93 (0.82-1.06)	0.271			0.94 (0.77-1.13)	0.488		
WBC count, ×10^9^/L	0.81 (0.75-0.88)	<0.001			0.72 (0.65-0.80)	<0.001		
Neutrophils count, ×10^9^/L	0.79 (0.71-0.87)	<0.001			0.61 (0.52-0.70)	<0.001		
Lymphocyte count, ×10^9^/L	0.91 (0.75-1.10)	0.315			0.79 (0.62-1.00)	0.049	1.42 (1.03-1.97)	0.035
Platelet count, ×10^9^/L	0.99 (0.98-0.99)	<0.001	0.99 (0.99-1.00)	<0.001	0.98 (0.98-0.98)	<0.001	0.99 (0.98-0.99)	<0.001
Hyaluronic, ng/ml	1.00 (1.00-1.01)	<0.001			1.01 (1.00-1.01)	<0.001		
Laminin, ng/ml	1.01 (1.01-1.02)	<0.001			1.01 (1.01-1.02)	<0.001		
PIIINP, ng/ml	1.06 (1.05-1.07)	<0.001			1.04 (1.03-1.04)	<0.001		
Type IV collagen, ng/ml	1.12 (1.10-1.13)	<0.001	**1.10 (1.08-1.12)**	**<0.001**	1.04 (1.04-1.05)	<0.001	**1.03 (1.02-1.04)**	**<0.001**

*NAFLD, non-alcoholic fatty liver disease; ALT, alanine transaminase; AST, aspartate aminotransferase; ALP, alkaline phosphatase; GGT, γ-glutamyl transpeptadase; TBil, total bilirubin; DBil, direct bilirubin; FBG, fasting blood glucose; TC, total cholesterol; TG, triglyceride; LDC, low-density lipoprotein; HDL, high-density lipoprotein; INR, international normalized ratio; APTT, activated partial thromboplastin time; PIIIP, N-Terminal procollagen III propeptide; APRI, AST to platelet ratio index; FIB-4, fibrosis-4; GPR, GGT to platelet ratio. The bold values are independent risk factors in the significant liver fibrosis group and liver cirrhosis*.

**Figure 2:**
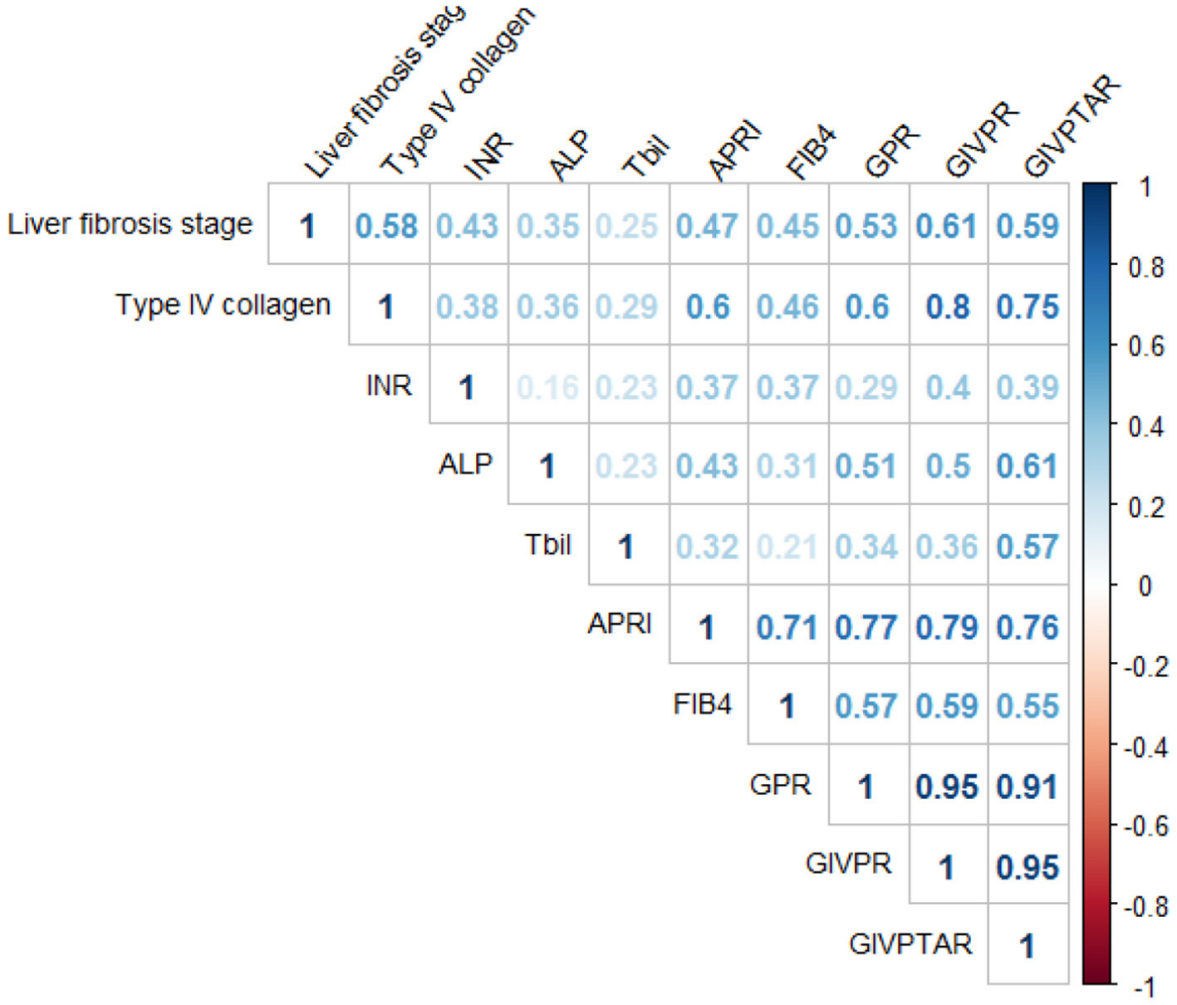
Correlation between the serum indexes and liver fibrosis score.

Spearman's correlation analysis showed that type IV collagen (*r* = 0.58), INR (*r* = 0.43), ALP (*r* = 0.35), and TBil (*r* = 0.25) were significantly correlated with liver fibrosis scores (Figure 2). Based on these independent predictors, we devised two simple models to amplify the predictive performances of the established non-invasive indices and serum parameters for the progression of liver fibrosis. The models are as follows: GIVPR = GPR × INR × type IV collagen; GIVPTAR = GPR × INR × type IV collagen × TBil × ALP. GIVPR (*r* = 0.61) was significantly positively correlated with the Scheure fibrosis score with a higher correlation coefficient than APRI, FIB-4, GPR, and GIVPTAR (*r* = 0.47, 0.45, 0.53, and 0.59, respectively) (Figure 2).

### Comparison of GIVPR With Other Non-invasive Indices for Predicting Liver Fibrosis in the Training and Validation Cohorts

**Figure 3:**
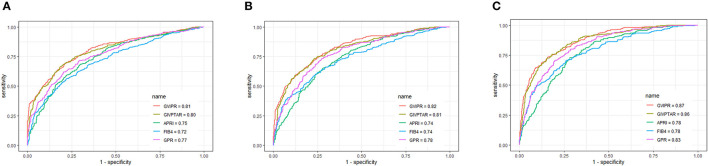
Area under receiver operating characteristic (ROC) comparison of GIVPR, GIVPTAR, APRI, FIB-4, and GPR in training set. **(A)** ROC comparison for predicting significant fibrosis; **(B)** ROC comparison for predicting advanced fibrosis; **(C)** ROC comparison for predicting cirrhosis.

**Figure 4:**
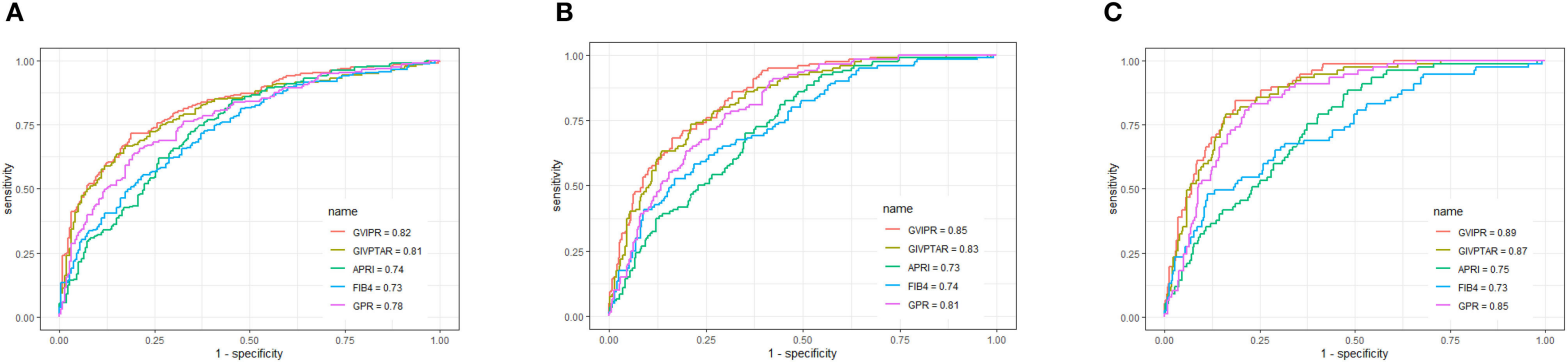
Area under receiver operating characteristic (ROC) comparison of GIVPR, GIVPTAR, APRI, FIB-4, and GPR in validation set. **(A)** ROC comparison for predicting significant fibrosis; **(B)** ROC comparison for predicting advanced fibrosis; **(C)** ROC comparison for predicting cirrhosis.

Using ROC curve analysis, GIVPR was compared to GIVPTAR, APRI, FIB-4, and GPR for staging liver fibrosis. GIVPR displayed better accuracy in predicting significant fibrosis, advanced fibrosis, and cirrhosis. The ROC curves for the fourth non-invasive serum marker are shown in the training set (Figure 3) and the validation set (Figure 4). In the training set, for the discrimination of significant fibrosis, GIVPR had the highest AUC (0.81, sensitivity 68.95% and specificity 79.23%) compared with GIVPTAR (0.80, sensitivity 69.53% and specificity 78.67%), APRI (0.75, sensitivity 68.37% and specificity 70.10%), FIB-4 (0.72, sensitivity 56.20% and specificity 77.86%), and GPR (0.77, sensitivity 71.37% and specificity 70.86%). When discriminating advanced fibrosis, GIVPR had the highest AUC (0.82, sensitivity 74.81% and specificity 74.57%) compared with GIVPTAR (0.81, sensitivity 75.06% and specificity 75.06%), APRI (0.74, sensitivity 65.14% and specificity 72.00%), FIB-4 (0.74, sensitivity 66.07% and specificity 70.38%), and GPR (0.78, sensitivity 73.03% and specificity 72.62%). For predicting cirrhosis, GIVPR also had the best AUC (0.87, sensitivity 73.33% and specificity 84.21%) compared with GIVPTAR (0.86, sensitivity 75.69% and specificity 81.80%), APRI (0.78 sensitivity 72.94% and specificity 71.86%), FIB-4 (0.78, sensitivity 70.59% and specificity 73.90%), and GPR (0.78, sensitivity 80.00% and specificity 71.44%). The cutoffs of GIVPR for the assessment of significant fibrosis, advanced fibrosis, and cirrhosis were 11.57, 15.45, and 29.07, respectively (Table 3).

**Table 3:** Predictive performances of GIVPR, GIVPTAR, APRI, FIB-4, and GPR for liver fibrosis in CHB patients (Training cohort).

**Indexes**	**AUROC (95%CI)**	**Cutoff**	**Se (%)**	**Sp (%)**	**PPV (%)**	**NPV (%)**	**Accuracy (%)**	^*^ ***P*** **-value**
**S2-4**								
GIVPR	0.81 (0.78-0.83)	11.57	68.95	79.23	81.3	66.1	73.41	–
GIVPTAR	0.80 (0.77-0.82)	11130.67	69.53	78.67	81.0	66.4	73.41	0.028
APRI	0.75 (0.72-0.77)	0.55	68.37	70.10	74.9	62.9	68.79	<0.0001
FIB-4	0.72 (0.69-0.75)	1.49	56.20	77.86	76.8	57.6	65.51	<0.0001
GPR	0.77 (0.74-0.79)	0.37	71.43	70.86	76.2	65.5	71.10	<0.0001
**S3-4**								
GIVPR	0.82 (0.80-0.85)	15.45	74.81	74.57	58.6	86.0	74.48	–
GIVPTAR	0.81 (0.79-0.83)	16667.88	75.06	75.06	59.1	86.2	75.06	0.008
APRI	0.74 (0.71-0.76)	0.78	65.14	72.00	52.8	81.1	69.61	<0.0001
FIB-4	0.74 (0.71-0.76)	1.49	66.07	70.38	51.7	81.2	68.73	<0.0001
GPR	0.78 (0.76-0.81)	0.52	73.03	72.62	56.2	84.9	72.75	<0.0001
**S4**								
GIVPR	0.87 (0.85-0.89)	29.07	73.33	84.21	55.3	92.2	81.92	–
GIVPTAR	0.86 (0.84-0.88)	32331.38	75.69	81.80	52.6	92.7	80.51	0.047
APRI	0.78 (0.75-0.80)	0.85	72.94	71.86	40.9	90.9	71.92	<0.0001
FIB-4	0.78 (0.76-0.81)	1.65	70.59	73.90	42.0	90.4	73.11	<0.0001
GPR	0.83 (0.81-0.85)	0.56	80.00	71.44	42.8	93.1	73.08	<0.0001

*AUROC, area under ROC; Se, sensitivity; Sp, specificity*.

* *Compared with GIVPR*.

Similarly, in the validation set, compared to the other four serum indices, GIVPR had the highest AUCs of 0.82 (sensitivity 73.82% and specificity 75.23%) for predicting significant fibrosis, 0.85 (sensitivity 81.67% and specificity 70.09%) for predicting advanced fibrosis, and 0.80 (sensitivity 84.42% and specificity 78.88%) for predicting cirrhosis (Table 4). These results suggest that GIVPR is an excellent predictor of liver fibrosis in CHB patients.

**Table 4:** Predictive performances of GIVPR, GIVPTAR, APRI, FIB-4, and GPR for liver fibrosis in CHB patients (Validation cohort).

**Indexes**	**AUROC(95%CI)**	**Cutoff**	**Se (%)**	**Sp (%)**	**PPV (%)**	**NPV (%)**	**Accuracy (%)**	^*^ ***P*** **-value**
**S2-4**								
GIVPR	0.82 (0.78-0.86)	11.57	73.82	75.23	76.0	72.6	74.28	–
GIVPTAR	0.81 (0.77-0.84)	11130.67	74.25	73.39	74.9	72.7	74.06	0.021
APRI	0.74 (0.70-0.78)	0.55	74.68	62.84	68.2	69.9	68.74	<0.0001
FIB-4	0.73 (0.69-0.77)	1.49	54.94	78.44	73.1	62.0	66.08	<0.0001
GPR	0.78 (0.73-0.81)	0.37	76.39	67.43	71.5	72.8	71.84	<0.0001
**S3-4**								
GIVPR	0.85 (0.81-0.88)	15.45	81.67	70.09	49.7	91.3	73.17	–
GIVPTAR	0.83 (0.79-0.86)	16667.88	80.00	69.18	48.5	90.5	71.84	0.006
APRI	0.73 (0.69-0.77)	0.78	59.23	74.31	71.1	63.0	65.41	<0.0001
FIB-4	0.74 (0.70-0.78)	1.49	65.00	70.69	44.6	84.8	68.96	<0.0001
GPR	0.81 (0.77-0.84)	0.52	77.50	69.79	48.2	89.5	71.62	<0.0001
**S4**								
GIVPR	0.89 (0.86-0.92)	29.07	84.42	78.88	45.1	96.1	80.27	–
GIVPTAR	0.87 (0.84-0.90)	32331.38	81.82	77.27	42.6	95.4	78.27	0.033
APRI	0.75 (0.70-0.79)	0.85	66.23	66.04	28.7	90.5	65.85	<0.0001
FIB-4	0.73 (0.69-0.77)	1.65	61.04	72.73	31.5	90.1	70.51	<0.0001
GPR	0.85 (0.81-0.88)	0.56	85.71	70.05	37.1	96.0	72.51	<0.0001

*AUROC, area under ROC; Se, sensitivity; Sp, specificity*.

* *Compared with GIVPR*.

### DCA for the Clinical Utility of GIVPR

**Figure 5:**
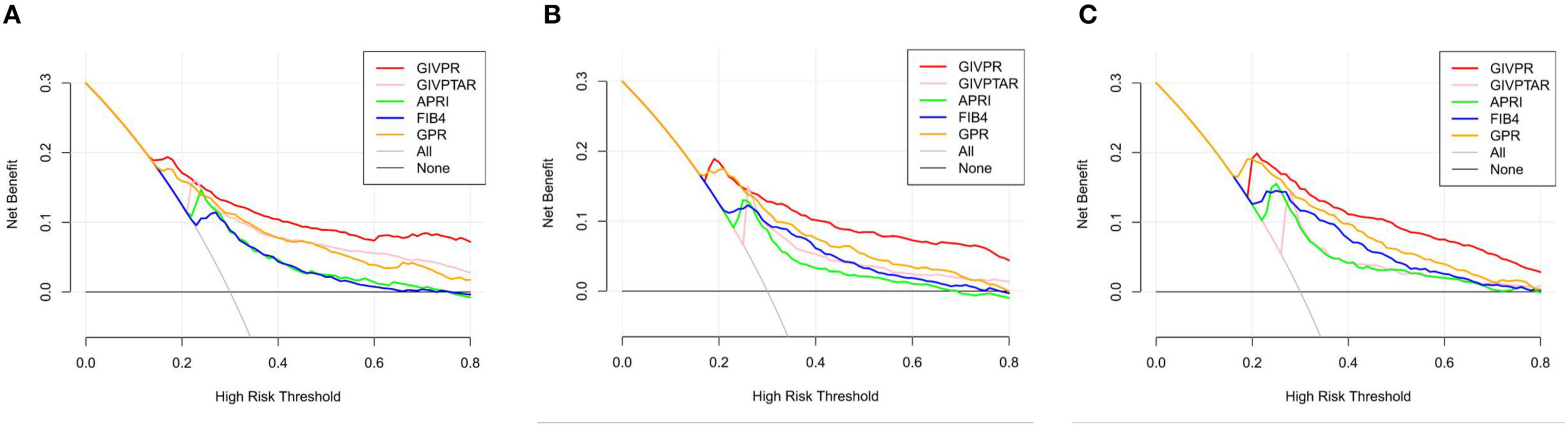
Liver fibrosis decision curve analysis in training set. Decision curve analysis depict the clinical net benefit. GIVPR is compared with GIVPTAR, APRI, FIB-4, and GPR for predicting significant fibrosis **(A)**; GIVPR is compared with GIVPTAR, APRI, FIB-4, and GPR for predicting advanced fibrosis **(B)**; GIVPR is compared with GIVPTAR, APRI, FIB-4, and GPR for predicting cirrhosis **(C)**. Black line, net benefit when no patient will experience the event; gray line, net benefit when all patients will experience the event. The preferred markers is the marker with the highest net benefit at any given threshold.

**Figure 6:**
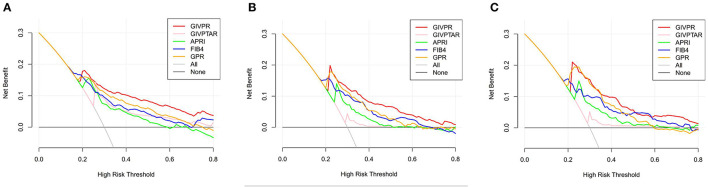
Liver fibrosis decision curve analysis in validation set. Decision curve analysis depict the clinical net benefit. GIVPR is compared with GIVPTAR, APRI, FIB-4, and GPR for predicting significant fibrosis **(A)**; GIVPR is compared with GIVPTAR, APRI, FIB-4, and GPR for predicting advanced fibrosis **(B)**; GIVPR is compared with GIVPTAR, APRI, FIB-4, and GPR for predicting cirrhosis **(C)**. Black line, net benefit when no patient will experience the event; gray line, net benefit when all patients will experience the event. The preferred markers is the marker with the highest net benefit at any given threshold.

Moreover, we conducted DCA to further investigate the clinical application value of GIVPR, GIVPTAR, APRI, FIB-4, and GPR for predicting liver fibrosis. In the training group, DCAs revealed that from a threshold probability of 20–80%, the application of GIVPR to predict liver fibrosis risk increased the benefit considerably more than the other four scores (Figure 5). Regarding the validation group, the DCAs of GIVPR also showed a better net benefit with a wide range of threshold probabilities and better performances for predicting liver fibrosis than GIVPTAR, APRI, FIB-4, and GPR (Figure 6).

## Discussion

Early diagnosis and accuracy in evaluating liver fibrosis or cirrhosis may play important roles not only in controlling disease progression but also in the treatment of chronic HBV infection (22). Liver biopsy is the gold standard for evaluating liver fibrosis in chronic liver disease. However, although liver biopsy is usually a safe procedure, it has some technical limitations and risks (23). Thus, there is an increasing need for simple and reliable non-invasive predictors for liver fibrosis, some of which have been evaluated in multiple studies. However, how their sensitivity and accuracy are affected by various factors is still a matter of debate (24). By combining non-invasive indicators, the overall diagnostic coincidence rate can be improved.

In the present study, we assessed the relationships between serum parameters and non-invasive indices and liver fibrosis in CHB patients. GIVPR and GIVPTAR based on GPR all exhibited excellent capacities to predict the progression of liver fibrosis. However, GIVPTAR, which required more variables, did not obtain higher AUCs than GIVPR and did not improve the predictive performance for liver fibrosis. We also compared the predictive accuracy of GIVPR with APRI, FIB-4, and GPR. Our results showed that in both the training and validation cohorts, GIVPR had the best AUC value for staging significant fibrosis, advanced fibrosis, and cirrhosis. Thus, GIVPR, which requires only GPR, INR, and type IV collagen and is simple to calculate, has a more powerful predictive performance for liver fibrosis in CHB patients.

There were two kinds of serum biomarkers for liver fibrosis progression, indirect serum markers and direct serum markers (25). Indirect serum markers had no direct correlation with liver fibrosis but reflected liver dysfunction or other fibrosis-related symptoms. They are often calculated into mathematical formulas or may be used individually (26). APRI and FIB-4 are the two non-invasive procedures for evaluating liver fibrosis that receive the most attention. They were reported to have a high AUROC to detect significant fibrosis and cirrhosis in CHB patients in East Africa and Asia (27, 28). The WHO CHB guidelines also recommend APRI and FIB-4 for application in resource-limited health care regions (29). However, a meta-analysis suggested that their diagnostic performance was not good enough to discriminate liver fibrosis in CHB patients and could not be used as an ideal replacement for liver biopsy (30). GPR is a novel index to assess liver fibrosis in patients with CHB in West African cohorts. It was shown to be better than the classical models APRI and FIB-4 (10). Additionally, GPR was reported to diagnose significant liver fibrosis and cirrhosis well in a large cohort of HBV monoinfected Gambian patients using FibroScan measures as a reference (31). However, GPR showed a less clear advantage in a Brazilian cohort and other Chinese cohorts (13, 32). In this study, our GIVPR model showed acceptable distinguishing power for the prediction of significant live fibrosis, advanced liver fibrosis, and cirrhosis in the training set, with AUCs of 0.797, 0.815, and 0.844, respectively; similar results were obtained in the validation set. Furthermore, we confirmed significantly better performance for the assessment of liver histological scores compared to the biochemical marker panels APRI, FIB-4, and GPR. Due to the different inflammatory and clinical conditions of patients with chronic hepatitis B and chronic hepatitis C, the effect of etiology on fibrosis progression and clinical biomarkers can explain this result (33, 34).

Moreover, the indirect serum markers evaluated in this study included the measurement of coagulation parameters, which were found to increase with the progression of liver fibrosis. Among these routine markers, INR was identified as an independent factor for the prediction of significant fibrosis and cirrhosis in CHB patients. Sterling et al. (21) reported that the INR was an independent predictor of liver fibrosis, and its concentration was directly related to liver function. Another study demonstrated that the INR level was associated with liver fibrosis and used INR as a parameter in their King's score, which was closely related to the progression of liver fibrosis (35, 36).

Direct biomarkers of liver fibrosis are fragments of liver matrix components produced in the process of fibrosis. These markers represent the intensity of fibrogenesis or fibrinolysis, such as type IV collagen, laminin, hyaluronic acid and metalloproteinases (37). Serum collagen levels, especially type IV, have been shown to be a useful, non-invasive measure of the activity of this pathway at a single time point and have been shown to reflect prognosis and responses to a variety of chronic liver diseases (14). Type IV collagen is an important component of the normal extracellular matrix. Compared with type I and type III collagen, which are partially hydrolyzed, type IV collagen remains intact in the matrix; therefore, the serum composition of type IV collagen is considered to mainly reflect the degradation of the matrix (38). Serum type IV collagen has been confirmed to be associated with both the progression of liver inflammation and fibrosis, which is in line with our data (26, 39).

This study has several limitations worth considering. First, this was a retrospective study in a single center and should be further confirmed in more patients from other centers. Second, GIVPR was not dynamically observed. We recommend further investigation into the efficacy of GIVPR compared to other non-invasive indices in evaluating fibrosis progression and in predicting liver-related end-stage disease after long-term antiviral inhibition of HBV.

## Conclusion

In summary, a novel non-invasive calculation, GIVPR, was established from GPR, INR, and type IV collagen. GIVPR demonstrates superior diagnostic accuracy and clinical usefulness compared to conventional serum indices. Although the clinical usefulness of GIVPR warrants future investigation, our findings showing that GIVPR is non-invasive and easily administered indicate that it could be a promising tool for the discrimination of liver fibrosis, especially in resource-limited regions.

## Data Availability Statement

The raw data supporting the conclusions of this article will be made available by the authors, without undue reservation.

## Ethics Statement

The study protocol and informed consent documents were reviewed and approved by the Ethics Committee of Shanghai Public Health Clinical Center, Fudan University. The patients/participants provided their written informed consent to participate in this study.

## Author Contributions

RD designed the study and wrote the manuscript. WL, XZ, and DH collected and analyzed data. YW and LY reviewed the statistical data. XL and WL were involved in critical revision of the paper. SS, ZZ, and LC approved the final manuscript. All authors have read and approved the manuscript.

## Funding

This work was supported by the 13th Five-year National Science and Technology Major Project of China (2017ZX10203202). This fund was acquired by ZZ. The funding body had no role in the design of the study and collection, analysis, and interpretation of data and in writing the manuscript.

## Conflict of Interest

The authors declare that the research was conducted in the absence of any commercial or financial relationships that could be construed as a potential conflict of interest.

## Publisher's Note

All claims expressed in this article are solely those of the authors and do not necessarily represent those of their affiliated organizations, or those of the publisher, the editors and the reviewers. Any product that may be evaluated in this article, or claim that may be made by its manufacturer, is not guaranteed or endorsed by the publisher.
